# Pandemic Shift: Virtual Self-Care Courses for Caregivers of Veterans

**DOI:** 10.1007/s41347-022-00294-y

**Published:** 2023-01-19

**Authors:** Jennifer Martindale-Adams, Deanna Stark, Jeffrey Zuber, Linda Scariano, April Green, Linda O. Nichols

**Affiliations:** 1 Department of Preventive Medicine, University of Tennessee Health Science Center, Memphis, USA; 2 Caregiver Center, VA Medical Center Memphis, Memphis, USA

**Keywords:** COVID-19, Caregivers, Veterans, Virtual

## Abstract

Self-care improves health and well-being, yet many caregivers neglect it. During COVID-19, self-care courses for caregivers of veterans transitioned from in-person to virtual videoconferencing. The format remained the same with caregiver groups and a trainer. This observational study examined in-person and virtual caregivers’ satisfaction with courses. Caregivers (1120 in-person, 962 virtual) could attend five courses before and following March 2020 transition to virtual. Evaluations (*N* = 1665) examined demographics, satisfaction, and utility. Characteristics were compared between in-person and virtual participants using chi-squared tests. Qualitative caregiver comments were compared. Half of the caregivers were over 60 years old; 49% had been caregivers at least 6 years. Caregivers were primarily women (91%) and spouses (75%), with more spouses virtually (*p* = 0.006) and more men in-person (*p* < 0.001). Both groups endorsed learning new information, planning to use it in caregiving and for themselves, increasing knowledge and skills, and having needs met. Caregiver comments revealed six types of benefits: new information, information review, positive effects, interaction, plans to act, and instructor qualities. Caregivers in virtual groups more often mentioned learning more information, being reminded of information, and planning to take further action; in-person caregivers more often mentioned interaction as a benefit. Caregivers were satisfied with and found benefit from in-person and virtual self-care courses. Although in-person courses allow for more social connection with others, virtual courses offer decreased travel costs for instructors and increased convenience and access for caregivers.

In 2015, about 34.2 million Americans provided care to an adult (National Alliance for Caregiving [NAC] & American Association of Retired Persons [AARP], 2004). Caregivers frequently neglect their own self-care (Acton, 2002; Salgado-Garcia et al., 2015), although self-care improves health and well-being (Oliveira et al., 2019; Pope et al., 2017; Waligora et al., 2019; Wang et al., 2019). Factors facilitating self-care include awareness and prioritization of the need for self-care, motivation to act, and knowledge of what to do (Kuhn et al., 2003; Oliveira et al., 2019; Waligora et al., 2019).

Self-care is generally understood to be activities to promote health, prevent disease, limit illness, and restore health (Lee et al., 2020; Levin, 1983). Training for caregivers has shown improved self-care. Improvements in measures of self-care, relaxation, sleep, fatigue, stress, self-rated health, and exercise have been shown for in-person courses with multiple sessions over multiple weeks (Powerful Tools for Caregivers; Building Better Caregivers) (Kuhn et al., 2003; Lorig et al., 2019). Online Building Better Caregivers training is extensively used in the VA and has shown benefit for pain, stress, and exercise (Lorig et al., 2012).

To meet caregivers’ needs for self-care, the Department of Veterans Affairs (VA) Caregiver Support Program (CSP) offers self-care courses to caregivers of veterans through the VA’s National Caregiver Center, located at the Memphis VA Medical Center. Before the COVID-19 pandemic, these courses were in-person for groups of caregivers affiliated with a VA health care facility. In mid-March 2020, VA travel was halted, and courses began to be offered virtually through videoconferencing. This observation study used previously collected data to determine if there were differences in caregivers’ satisfaction between virtual and in-person courses for the year before and after March 17, 2020.

## Methods

### Self-Care Courses

There were five stand-alone courses available before and after the pandemic started. For each, a group of caregivers from a facility participated. For in-person, to maximize trainer travel, a facility would request two different courses at a time (e.g., managing stress and handling emotions) to be offered on the same day. For virtual, a facility would request a course to be offered on a specific day. To increase the number of caregivers attending, facilities could also join another facility to participate in the course. Facilities chose which courses would be offered to their caregivers. Caregivers who were participants in any CSP programs could participate. Any facility could request any course, and caregivers could attend any course(s). English and Spanish courses were taught. An instructor from the VA Caregiver Center at the Memphis VA Medical Center, plus Spanish-speaking contractors, taught each course. Each caregiver received a workbook. Virtual caregivers received an email link to a videoconference platform to view the course slide deck and interact with the instructor and each other.

The courses are grounded in theory. In stress health-process theory (Cohen et al., 1995; Lazarus & Launier, 1978), caregivers experience stress if they perceive their demands, including external challenges and their responses, are greater than their resources. In addition to managing emotions, self-efficacy theory (Bandura, 1977; Maddux, 1995) supports caregivers’ belief in their ability to succeed through the previous accomplishment of similar tasks, observing others, and visualizing successful performance. Thus, courses provide information, discussion, resources, a caregiver workbook, and support. Skills are taught, such as problem-solving to manage demands and cognitive reframing and stress management to manage responses to demands. Courses provide time to practice new skills and an opportunity to reflect on how past experiences link to new skills.

Content and topics were the same for in-person and virtual courses, although in-person courses tended to be 3 h rather than 2, with longer caregiver introductions and breaks with more caregiver interaction with each other, the trainer, and facility staff present. Courses include:

- Problem-solving and effective communication—problem-solving approach; communication principles; practice
- Managing stress—sources of stress; the practice of touch, stretching, deep breathing, meditations, journaling
- Lowering stress, improving mood—the practice of yoga, music, qigong, mindful doodling, and mood management
- Taking care of your physical health—healthier lifestyle and illness prevention through rest, nutrition, and exercise; relaxation practice; VA resources: information, respite
- Handling emotions—definition, impact, purposes of emotions; common myths and beliefs; healthy ways of dealing with emotions; emotional balance.

### Measures

For each course, caregivers were asked to fill out a paper evaluation form. Pre-pandemic, these were collected in-person; for virtual, individual registered caregivers were sent a workbook, an evaluation form, and post-paid envelope to return their evaluation. The evaluation form is included as a supplemental file to this article. The evaluation was overseen by the Memphis VA Medical Center Institutional Review Board.

Collected demographic information included relationship, sex, age, and time as a caregiver. To assess satisfaction and utility parameters, on a Likert scale from strongly disagree (1) to strongly agree (5), caregivers were asked about the instructor and if they would recommend the course, whether the content met needs, and whether they learned new information, increased knowledge and skill as a caregiver, and planned to use the information in their caregiving and for themselves to take care of physical and emotional health. Caregivers were invited to say what topic was most and least useful and to provide additional comments.

### Analysis

Characteristics were compared between in-person and virtual participants using chi-squared tests. *P* values ≤ 0.05 were considered statistically significant. Cramer’s *V*, used to estimate effect size, is interpreted as the strength of the relationship between two nominal variables; it ranges between 0 and 1 (a higher value indicates a stronger relationship). Values of 0.1, 0.3, and 0.5 have been characterized as small, medium, and large effects respectively (Cohen, 1988). Courses were compared to determine if facilities requested different in-person and virtual courses. For qualitative analysis, responses were first examined individually by one author with prior experience in coding of qualitative data to sort descriptions, concepts, and central ideas into potential themes (Bernard, 2017). Two other authors reviewed initial themes to determine saturation with no new codes occurring (Urguhart, 2013). All three finalized themes and linked them to quotes (Bernard, 2017).

## Findings/Results

There was no significant difference in the courses facilities requested to offer to their caregivers during the in-person and virtual periods. For example, problem-solving represented 25% of total in-person courses requested and 22% of virtual courses requested. For the five courses available, 1120 caregivers participated in-person in the year prior to March 17, 2020, with 962 participating virtually in the year after. Evaluations were filled out by 1080 caregivers who attended in-person and 585 caregivers who attended virtually, return rates of 96% and 61%, respectively.

**Fig. 1:**
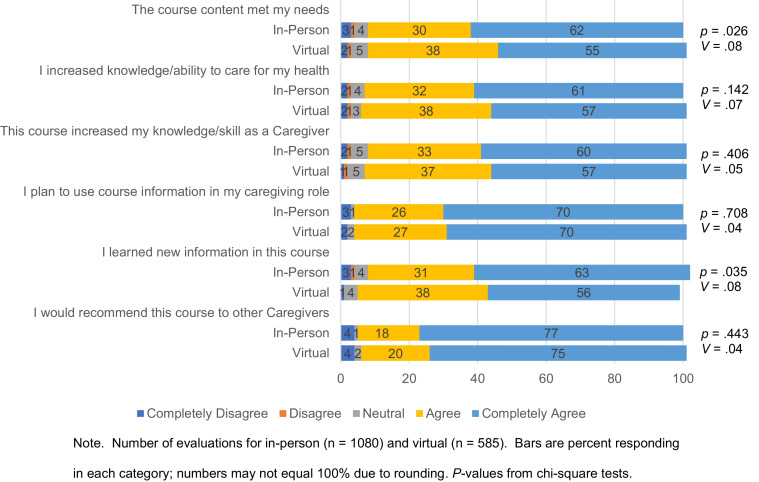
Caregiver responses for satisfaction and utility parameters of in-person and virtual courses

Caregivers were primarily women (91%) and spouses (75%). About half were at least 60 years old (50%) and had been caregivers at least 6 years (49%). There were two significant demographic differences between groups, with more spouses attending virtually and more men in-person (Table 1), although effect sizes were small. For satisfaction and utility parameters, as shown in Fig. 1, caregivers in in-person and virtual courses similarly reported they learned new information, increased knowledge, and caregiving skills and would use the information in their caregiving. There were two significant differences between groups—in-person caregivers more often completely agreed content met their needs and they would use the information for self-care. Effect sizes were again small.

**Table 1:** Caregiver demographics

Factor	In-person, % (*n* = 1080)^a^	Virtual, % (*n* = 585) ^a^	*p* value^b^	Cramer’s *V*
Relationship to Veteran			0.006	0.10
Spouse/partner	72.7	80.2		
Child	12.4	9.0		
Parent	6.3	6.1		
Friend	2.1	0.9		
Other	6.5	3.8		
Sex			< 0.001	0.09
Female	89.5	94.6		
Male	10.5	5.4		
Age			0.716	0.05
19 or younger	0	0		
20–29 years	1.1	0.5		
30–39 years	10.6	12.2		
40–49 years	16.7	17.2		
50–59 years	20.5	20.8		
60–69 years	26.4	25.7		
70–79 years	19.1	19.4		
80 + years	5.6	4.2		
Years caregiving			0.077	0.07
Less than 1 year	6.0	3.5		
1–2 years	14.4	14.2		
3–5 years	32.8	29.5		
6–10 years	22.5	24.9		
More than 10 years	24.3	27.9		

^a^Number of returned evaluations

^b^*p*-values from chi-square tests

In qualitative analysis, many caregivers were not specific in their comments, e.g., “*Liked it all*.” (participant 11, in-person attendee; participant 971, virtual attendee). However, caregivers in both groups reported six specific types of benefits:

- new information, including general and specific information and self-awareness, e.g., *“I resonated with the fact that I experience [ambiguous] loss every time my veteran slips or relapses after a success.”* (participant 623, in-person attendee); “*How common depression is for caregivers was very useful. That stresses the importance of managing your emotions*.” (participant 186, virtual attendee);
- reminder/review of known information/skills, e.g., *“reinforced benefits of stress relief measures I was aware of but not following through with.”* (participant 938, in-person attendee)*;* “*Most of it was reminding me of what I've learned over the years. I need reminders. I must be reminded to have positive thoughts and avoid negative news and negative people as much as possible, but to prepare myself emotionally ahead of time if I have to be around negative people*.” (participant 185, virtual attendee);
- positive effects from attending, e.g., “*Just the [meditation] exercise I did here caused the anxiety in my stomach to go away – at least for about 30 min.*”) (participant 839, in-person attendee); “*Being refreshed by the information presented. Also to know that it is okay to feel angry and frustrated without the guilt*.” (participant 213, virtual attendee);
- interaction with others, e.g., “*Meeting others & listening to what they go through and knowing you are not the only one that feels the way you do.*” (participant 890, in-person attendee); “*I want to thank you so much for helping us caregivers in knowing that we are not alone*.” (participant 904, virtual attendee);
- plans to take further action, e.g., “*The idea of starting a blog has been rolling around in my head for a while. Think I will act.*” (participant 1064, virtual attendee); “*Taking personal action–what thing can I do for me?*” (participant 898, in-person attendee); and
- instructor qualities, e.g., “*Information was provided in a non-lecture way. It was wonderful, and [the] presenter made it fun”* (participant 779, in-person attendee); “*This class is wonderful & needed. What makes it even more wonderful is the loving instructors. God bless all who work for & with the VA.*” (participant 196, virtual attendee).

There were some differences between the groups. Caregivers in virtual groups more often mentioned learning more information (“*I didn't know some of my triggers to stress. The negative self-talk is a big one. Also learning about ‘freeze’ during a stressful experience. I also learned new techniques to relieve stress*.”—participant 1014, virtual attendee), being reminded of information (“*Also, the explanation of fight, flight or freeze reactions brought to mind many examples of each in my own experience*”—participant 1042, virtual attendee), and planning to take further action (“*I have not done Qigong before so that was a great introduction. I will be using those techniques as well as the yoga stretches regularly”*—participant 274, virtual attendee). In-person caregivers more often mentioned interaction (“*enjoyed the interaction with the rest of the group*”—participant 11, in-person attendee). While virtual caregivers mentioned interaction also, they also mentioned the lack of interaction, “*Personally I prefer in person training as it's easier to ask questions. However, with this said it's better than not having the training.*”—participant 907, virtual attendee). For virtual caregivers, a small number reported some technical difficulty, ranging from missing sound or audio to not knowing how to ask questions, (“*Even with the technical problems today, the situation was fixed and class continued. Most would have ended it but you didn't give up*.”—participant 903, virtual attendee).

## Discussion

When the COVID-19 pandemic occurred, the VA’s in-person self-care courses for caregivers of veterans became virtual. In comparing the two types of courses, caregivers who participated were similar, and their satisfaction with the courses was similar. Benefits identified by both types of caregivers included learning new information, reminder/review of known information/skills, positive effects from attending, interaction with others, plans to take further action, and instructor qualities. These similar benefits suggest the courses were meeting similar needs.

There were some differences between the two groups. More men participated in-person; more spouses participated virtually. More caregivers in-person, compared to virtual, strongly agreed content met their needs, and they would use the information for their own care. Caregivers in virtual groups more often mentioned learning new information, reviewing known information, and planning to take action as benefits, while in-person caregivers more often mentioned interaction with other group members as a benefit of the training. Two findings were more closely related to the type of interaction—virtual vs. in-person. A small number of virtual caregivers reported technology concerns, which would not have been an issue for in-person caregivers. While virtual caregivers mentioned interaction as a benefit, more in-person caregivers valued the social contact with other group members as a benefit. With multiple participants on a conference call format and limited opportunity for socializing during breaks, virtual training courses may not be able to replicate this important benefit.

There are some study limitations. The number of demographic variables collected was relatively small. There was a lower evaluation return rate for virtual courses, although this rate is consistent with mailed survey return rates (Dillman et al., 2014). Another possible limitation is caregivers who were not comfortable with technology may have declined to participate in the virtual courses. The similarity in demographics between the two groups suggests the sample is representative of the caregivers available to attend. In addition, caregivers in this sample are like caregivers of veterans nationally. Caregivers of veterans nationally are most often spouses (70%) and women (96%) with 56% providing care for more than 5 years (NAC, 2010). For this sample, 75% were spouses, and 91% were women with 49% providing care for at least 6 years. Caregivers of veterans nationally have 61% over age 50 (NAC, 2010); for this sample, 71% were over age 50.

Several factors facilitated the success of the virtual courses. The VA ramped up quickly to provide secure platforms for large-scale trainings. Instructors edited instructions automatically sent by the virtual platform to be clear and simple and were on early to help participants who were not as technologically savvy. To simplify the process for caregivers, decrease cost and staff time, and increase the rate of return, evaluations are now being collected through a link sent after the course. Finally, caregivers’ growing familiarity with virtual technology has provided opportunities for expansion.

Caregivers’ embrace of the five virtual self-care courses and their reports of the isolation and challenges of caregiving during the pandemic spurred the development of additional types of courses to help them cope. Occasional courses on topics of interest, such as intimacy and taking care of yourself during the holidays, publicized nationally instead of being limited to individual VA facilities, were developed. During the pandemic’s first year (March 2020–March 2021), 308 caregivers attended four open courses; during the second year (March 2021–March 2022), 914 caregivers attended nine open courses.

Three wellness series were also developed, each consisting of three short (less than 1 h) courses delivered by telephone and/or virtually. The Resilience series includes Self-discovery, Self-compassion, and On-the-job self-care. Wellness through Qigong series includes on Slow, gentle movements, Self-applied massage, and Breathwork. There are also three mindfulness courses, Paying attention, On purpose, and Without judgment. These courses, which typically do not have accompanying materials other than an emailed handout, have been popular with caregivers, with 610 caregivers participating during the pandemic’s first year and 1652 during the second. Evaluation is ongoing.

Despite fears virtual interactions would be challenging and impersonal, caregivers, including older caregivers, have been satisfied with and found benefit from virtual self-care and in-person courses. Although there were differences between groups, neither in-person nor virtual courses were favored overall in terms of satisfaction and benefit. Because instructors’ time investment is less without travel, courses can be offered to smaller groups of caregivers, and sites can partner to increase the number of caregivers in a course. As instructors have become more comfortable with this expanded virtual environment, newer courses, such as qigong, focus more on interaction and movement than slides and didactic instruction. As caregivers become more comfortable with virtual interaction, specific ways to stimulate informal discussion and connection should be explored, perhaps during planned breaks and activities. Although the original plan was to return to in-person courses post-pandemic, the success of virtual courses means more caregivers can be reached with less cost.
